# Neon Pink Breast Milk Stain in the NICU

**DOI:** 10.7759/cureus.78425

**Published:** 2025-02-03

**Authors:** Brenda Stanley, Pratik Parikh

**Affiliations:** 1 Neonatology, Christus Children's Hospital, San Antonio, USA; 2 Neonatology, Baylor College of Medicine, San Antonio, USA

**Keywords:** breast milk bacterial transmission, breast milk contamination, neonatal intensive care unit (nicu), neonatal sepsis, serratia marcescens

## Abstract

Pink discoloration in breast milk is a rare phenomenon arising from various benign or pathological causes. Serratia marcescens, a gram-negative bacterium known for its characteristic red pigment prodigiosin, is a notable cause. This case report examines the clinical approach, diagnostic workup, microbiological findings, and management strategies for a neonate presenting with neon pink stains in breast milk.

A female neonate born at 30+5 weeks gestation via repeat cesarean section was admitted to the neonatal intensive care unit (NICU). On day of life (DOL) 28, bright pink staining was observed in expressed breast milk (EBM) and secretions. Initial management included a septic workup, temporary cessation of feeds, and cultures of breast milk and fortified breast milk. The infant was started on piperacillin-tazobactam empirically while awaiting culture results. Blood cultures were negative, but cultures of breast milk and tubing showed heavy growth of S*erratia marcescens*, *Klebsiella oxytoca*, and *Pseudomonas *species. Feeds were resumed with Similac Special Care 24 kcal/oz, and the mother was treated with ciprofloxacin for seven days. Repeat cultures of breast milk showed no gram-negative bacterial growth.

*S. marcescens*, an opportunistic pathogen, can cause pink discoloration in breast milk due to its pigment prodigiosin. The bacterium is associated with hospital-acquired infections, particularly in NICUs, where it thrives in moist environments and can contaminate breast pump equipment and feeding utensils. Rigorous infection control measures, including proper sterilization and handling of breast milk, are essential in preventing outbreaks.

This case highlights the importance of thorough investigation and prompt management of unusual findings such as pink breast milk. Adhering to stringent hygiene and infection control protocols is critical in ensuring the safety of neonates and the breastfeeding process.

## Introduction

Breast milk is widely recognized as the optimal source of infant nutrition, providing essential nutrients and antibodies and fostering a unique bond between mother and child. Under normal circumstances, breast milk appears white or yellowish, occasionally exhibiting variations in hue due to dietary factors, medications, or maternal health [1]. However, the appearance of pink or neon pink breast milk is an unusual and alarming phenomenon that necessitates immediate investigation to rule out potential health risks to the infant.

Pink discoloration in breast milk can be attributed to several causes, including blood contamination, certain foods, medications, or, in rare cases, bacterial contamination. While benign causes such as maternal nipple trauma or the ingestion of certain foods like beets or artificially colored drinks are common, the possibility of bacterial contamination poses a significant health risk, particularly in neonates and immunocompromised infants.

One bacterium of particular concern is *Serratia marcescens*, known for its characteristic red pigment, prodigiosin, which can give breast milk a pinkish hue [2]. *S. marcescens* is an opportunistic pathogen frequently associated with hospital-acquired infections, especially in NICUs. Its presence in breast milk can lead to serious infections, including sepsis, necessitating prompt and effective intervention.

The phenomenon of pink breast milk, while rare, underscores the importance of vigilant monitoring, proper hygiene, and handling practices to prevent contamination and ensure the safety of both the breastfeeding process and the infant [3]. This case report delves into an incident of neon pink staining in breast milk, exploring the clinical approach, diagnostic workup, microbiological findings, management strategies, and relevant literature to provide a comprehensive understanding of this unusual presentation.

## Case presentation

The patient is a female neonate, born at 30+5 weeks gestation via repeat cesarean section. Initial APGAR scores were 8/8. She was admitted to the NICU on continuous positive airway pressure (CPAP) +6 at 30%. Birth weight was 1030 grams (12th percentile). Vital signs were as follows: temperature 98.2°F, heart rate (HR) 139, respiratory rate (RR) 38, blood pressure (BP) 45/23 (32), and oxygen saturation 100%.

The mother is a 33-year-old G3P3 with chronic hypertension and superimposed preeclampsia. Rapid plasma reagent (RPR) was reactive, with treatment administered earlier in pregnancy. Fetal intrauterine growth restriction (IUGR) was suspected, with fetal growth in the 5th percentile.

Initial management included NPO with total parenteral nutrition (TPN) (D10) via an umbilical venous line. The initial complete blood count (CBC) showed an absolute neutrophil count (ANC) of 572. The infant's RPR, which is a test for syphilis, was reactive with a titer of 1:8. Urine CMV testing, conducted due to low ANC and small for gestational age (SGA) status, was negative. Head and abdominal ultrasounds (US) were normal. Maternal RPR titers on admission were not fourfold higher than those at the end of treatment, ruling out re-infection. The infant received a single dose of benzathine penicillin G.

Maternal breast milk feeds were initiated and advanced as tolerated. The mother declined donor breast milk use. Expressed breast milk (EBM) was fortified to 26 kcal/oz to support growth. Oral feeding attempts were initiated when the feeding readiness scores indicated support.

**Figure 1:**
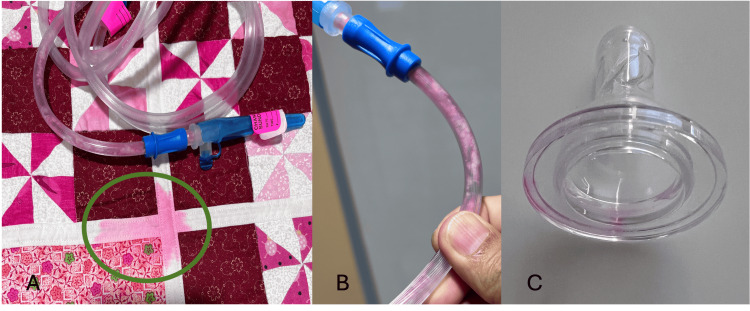
(A) Neon pink stains on the blanket. (B) Neon pink secretion stains. (C) Neon pink discoloration of the nipple.

At 28 days of age, bright pink secretions were observed in the oral suction tubing, and the bedding also had bright pink stains (Figure 1). The neonate remained alert and active with no clinical signs of sepsis. The initial differential diagnoses included oral injury, gastritis, gastric injury, and sepsis. No oral injury or fresh blood was noted in the oral cavity.

The infant underwent a diagnostic workup that included a complete blood count, oral aspirate and blood cultures, abdominal X-ray, and bacterial cultures of breast milk, fortified breast milk, and formula. Infant feeds were discontinued, and intravenous fluids were started. Empiric antibiotic therapy with piperacillin-tazobactam was initiated. An infectious disease physician was consulted.

Blood cultures were negative. Breast milk culture and oral aspirate culture were positive for *S. marcescens*, *K. oxytoca*, and *Pseudomonas* species. The formula fluid culture was negative.

The infant continued to show no signs of sepsis, and feeds were reintroduced with Similac Special Care 24 kcal/oz. Due to positive breast milk culture, and the per infectious disease recommendation, the mother was treated with ciprofloxacin for seven days. Repeat breast milk cultures showed no gram-negative bacterial growth. The infant was discharged home after a few days on oral feeds of breast milk and formula.

## Discussion

*S. marcescens* is a significant concern in NICUs due to its ability to cause serious infections, including sepsis, in highly vulnerable infants. This opportunistic pathogen is commonly found in healthcare environments and can contaminate various surfaces and devices, such as medical equipment, respiratory devices, and breast pumps. Contamination of EBM with *S. marcescens* has been implicated in several NICU outbreaks, resulting in infections that can manifest as bacteremia, meningitis, and other serious illnesses [4]. The first recorded case of *S. marcescens* colonization dates back to 1958 when Waisman and Stone described “red diaper syndrome” in an infant whose diapers displayed a red discoloration caused by the bacterium [5]. This initial case highlighted the potential for contamination to occur from external sources, as the bacterium was traced back to a research lab near the infant’s home.

In hospital settings, *S. marcescens* can be found in breast pumps and other equipment, leading to contaminated EBM. Infections have been reported in infants after exposure to EBM with high levels of bacterial growth, sometimes reaching over 1,000,000 colony-forming units (CFUs) per milliliter, which has been associated with cases of neonatal sepsis. For instance, one report detailed a term infant who developed *S. marcescens* bacteremia at 17 days of age after consuming contaminated breast milk during hospitalization. The strain involved in that case did not make the characteristic red pigment known as prodigiosin, which is sometimes produced by *S. marcescens* strains at temperatures below 37°C [6].

The level of contamination that can cause harm to infants remains uncertain. Some studies suggest that contamination above 1,000 CFU/mL may lead to feeding intolerance in preterm infants, while levels exceeding 1,000,000 CFU/mL may cause systemic infections [7]. While recommendations for managing *S. marcescens* contamination vary, interventions can range from counseling families on proper milk-handling techniques to temporarily discarding affected EBM and administering antibiotics to the infant, mother, or both. Although breast pump cleaning is a crucial practice to prevent contamination, evidence regarding its effectiveness in eliminating *S. marcescens* colonization or preventing recurrence after treatment is limited [8].

Routine bacterial contamination of EBM is common, but improving milk collection and storage practices has been shown to reduce bacterial counts. The CDC has issued guidelines for breast pump cleanliness to help decrease the risk of contamination, which is particularly important for parents of NICU infants who rely on expressed milk [3]. While treating asymptomatic colonization in otherwise healthy infants is not generally supported due to the risks of adverse reactions and antibiotic resistance, healthcare providers need to recognize the possibility of *S. marcescens* in cases of "pink milk." Awareness and adherence to best practices in EBM handling are critical to safeguarding the health of infants in NICUs.

## Conclusions

The occurrence of pink breast milk due to *S. marcescens* contamination highlights the need for vigilant monitoring, thorough investigation, and prompt management. Adhering to stringent hygiene and infection control protocols is essential to minimize contamination and ensure the health and safety of neonates, particularly in NICUs. Early detection and intervention are critical to preventing severe infections and ensuring the well-being of both the infant and the breastfeeding process. Healthcare providers play a crucial role in supporting continued breastfeeding while managing the risks associated with bacterial contamination.
